# Patient preferences for treatment modalities for localised prostate cancer

**DOI:** 10.1002/bco2.198

**Published:** 2022-11-17

**Authors:** Frederik R. Teunissen, Charisma Hehakaya, Richard P. Meijer, Harm H. E. van Melick, Helena M. Verkooijen, Jochem R. N. van der Voort van Zyp

**Affiliations:** ^1^ Department of Radiation Oncology University Medical Center Utrecht Utrecht The Netherlands; ^2^ Imaging and Oncology Division University Medical Center Utrecht Utrecht The Netherlands; ^3^ Department of Oncological Urology University Medical Center Utrecht Utrecht The Netherlands; ^4^ Department of Urology St. Antonius Hospital, Nieuwegein Utrecht The Netherlands; ^5^ Utrecht University Utrecht The Netherlands

**Keywords:** localised prostate cancer (PCa), treatment decision making, treatment outcomes, treatment preferences, utility scores

## Abstract

**Objectives:**

To assess the patient preferences and utility scores for the different conventional and innovative treatment modalities for localised prostate cancer (PCa).

**Subjects and Methods:**

Patients treated for localised PCa and healthy volunteers were invited to fill out a treatment‐outcome scenario questionnaire. Participants ranked six different treatments for localised PCa from most to least favourable, prior to information. In a next step, treatment procedures, toxicity, risk of biochemical recurrence and follow‐up regimen were comprehensibly described for each of the six treatments (i.e. treatment‐outcome scenarios), after which patients re‐ranked the six treatments. Additionally, participants gave a visual analogue scale (VAS) and time trade‐off (TTO) score for each scenario. Differences between utility scores were tested by Friedman tests with post hoc Wilcoxon signed‐rank tests.

**Results:**

Eighty patients and twenty‐nine healthy volunteers were included in the study. Before receiving treatment‐outcome scenario information, participants ranked magnetic resonance‐guided adaptive radiotherapy most often as their first choice (35%). After treatment information was received, active surveillance was most often ranked as the first choice (41%). Utility scores were significantly different between the six treatment‐outcome scenarios, and active surveillance, non‐ and minimal‐invasive treatments received higher scores.

**Conclusions:**

Active surveillance and non‐invasive treatment for localised PCa were the most preferred options by PCa patients and healthy volunteers and received among the highest utility scores. Treatment preferences change after treatment information is received.

## 1 INTRODUCTION

The majority of prostate cancer (PCa) patients have localised disease at the time of diagnosis. Overall survival rates are high due to the nonaggressive nature of many localised prostate tumours and the effective treatment options.^1^ Treatment modalities for localised PCa include external beam radiotherapy (EBRT), low‐dose‐rate (LDR) brachytherapy (BT), robot‐assisted radical prostatectomy (RARP) and for low‐risk PCa, active surveillance (AS).^2^ Radical treatments bear a considerable risk of adverse effects such as erectile dysfunction, urinary problems and bowel problems. New treatment modalities that aim to reduce adverse events are being developed, such as magnetic resonance‐guided adaptive radiotherapy (MRgRT) and focal therapy (FT), including irreversible electroporation, high‐intensity focused ultrasound and cryoablation, but long‐term functional and oncological outcomes are not yet available.^3^, ^4^, ^5^, ^6^

Differences in risk for adverse events between the different treatment modalities are often poorly understood by patients.^7^ It is reported that 65% of the patients do not know that they are at greater risk for incontinence after RARP than after radiotherapy (i.e. EBRT and BT) and that 61% have a comparable wrong perception about erectile dysfunction. On the other hand, 53% of the patients were unaware that after RARP, the risk for bowel problems is lower than after radiotherapy. Furthermore, 80% do not understand that mortality rates are comparable following AS, RARP, EBRT and BT. Also, the risk of requiring definitive treatment after AS is sometimes overestimated by patients. Often, a patient's decision to receive active treatment versus AS is largely based on the urologist's recommendation.^8^

Besides treatment preference, cost‐effectiveness is an important factor in the evaluation of conventional treatments and the implementation of new treatments. New, and often costly, innovative treatments are often rushed into clinical standard care before treatment superiority is proven against conventional treatments.^9^ Early health technology assessment (HTA) aims to evaluate the cost‐effectiveness of new treatments at an early stage. Utility scores, which are the measures of value that an individual gives under conditions of uncertainty that satisfy certain aspects, can guide in the early HTA of new treatment strategies for both conventional and new treatment modalities.^10^

It remains unclear what treatment modalities for localised PCa are preferred by patients in case multiple treatment options are available, and what utility scores patients would give to those treatments. We aimed to assess the patient preferences and utility scores for treatment modalities for localised PCa.

## 2 SUBJECTS AND METHODS

### 2.1 Participants

The study was approved by our institutional ethics review board. Included were patients participating in the ‘Utrecht Prostate Cohort’ (NCT04228211), who signed informed consent for receiving questionnaires and participating in future studies.^11^ An unselected consecutive sample of equal numbers of patients who underwent AS, RARP, conventional EBRT and MRgRT were invited to participate in the present study. Patients who underwent treatment were approached at least six months after treatment.

Patients were invited by email and participating patients were asked to invite up to a maximum of five healthy male volunteers without PCa, over 50 years of age, to anonymously fill out the same questionnaire. Questionnaires were filled out online through a secured link. Patients and volunteers were encouraged to fill out the questionnaires individually.

### 2.2 Treatment‐outcome scenario questionnaire

A comprehensive treatment‐outcome scenario questionnaire was developed based on the literature. The developed questionnaire was reviewed by an epidemiologist, a radiation oncologist and two urologists (Supporting Information S3).

Firstly, participants were asked to fill out a general questionnaire including questions on patient characteristics and self‐assessed health by the validated five‐level version of the EuroQol 5‐dimension (EQ‐5D‐5L) questionnaire.^12^

Secondly, participants were asked to hypothesise being newly diagnosed with localised PCa and eligible for the following six treatment options: AS (no active treatment), RARP, conventional EBRT receiving 5 × 7.25Gy, LDR BT receiving radiation via interstitial iodide‐125 sources, MRgRT receiving 5 × 7.25Gy on an MR‐Linac and FT by irreversible electroporation. Each treatment was described in one sentence, after which participants were asked to rank treatment options from most to least favourable.

Thirdly, treatment‐outcome scenarios for all six treatment options were extensively described, including treatment procedures, and possible complications and adverse effects, along with their probability based on the European PCa guidelines and recent literature (Supporting Information S1).^1^, ^13^, ^14^, ^15^, ^16^, ^17^, ^18^, ^19^ Prognosis was described as the chance of biochemical recurrence and PCa‐specific mortality risk within 10 years after treatment. For the assessment of utility scores, participants were asked to rate each scenario on a visual analogue scale (VAS) from 0 to 100. A score of 0 represented the treatment‐outcome scenario as the worst thinkable health condition and a score of 100 represented the treatment‐outcome scenario as perfect health. Additionally, participants were asked to rate the scenarios according to the time trade‐off (TTO) method. With the TTO method, participants indicate how many years (with a range of 0 to 10) of life in perfect health weigh up to 10 years of life in the health status after treatment as described in the specific scenario. A score of 0 indicated living with the outcomes of the treatment‐outcome scenario to be unbearable and a score of 10 as equal to living in perfect health. To improve and estimate the participants' understanding of the scoring methods, the questionnaire contained two scenario examples and two exercise scenarios to start with.

Fourthly, the question to rank the six given treatment options from most favourable to least favourable was repeated, but now with the aforementioned background information from the six treatment‐outcome scenarios. All four steps were on separate subsequent electronic form pages and participants were urged not to go back.

The treatment‐outcome scenario questionnaire was tested in a pilot study with five PCa patients. Their comments were used to improve the questionnaire.

### 2.3 Statistical analysis

The ranks of the six treatment options were presented as proportions. Proportions (discrete variables) and medians with interquartile range or means with SD (continuous variables) were calculated for baseline characteristics and VAS (0–100) and TTO (0–10) scores. The Spearman's rank correlation coefficient (ρ) was calculated to assess the correlation between VAS and TTO scores. We used the Friedman test to compare VAS and TTO scores between treatment scenarios. Stratified analyses were performed to evaluate whether utility scores differed for patients and volunteers, by age, previous PCa treatment, education and baseline EQ‐5D VAS score. When the Friedman test found a significant difference between the six ratings, the AS scenario score was compared to all the other five scenario scores using post hoc Wilcoxon signed‐rank tests. Bonferroni corrections for multiple testing were performed for all tests. The level of significance was set at *p*< 0.05. Statistical analysis was performed using R version 4.0.5.

## 3 RESULTS

The questionnaire was sent out to 124 patients. Twenty‐one (68%) AS, 21 (68%) MRgRT, 21 (68%) conventional EBRT and 17 (55%) RARP patients completed the questionnaire. The median time between treatment or start of AS and filling out the questionnaire was 7.3 months (range: 6.0–10.0). At time of filling out the questionnaire, no patient had experienced tumour recurrence. Thirty‐six volunteers completed the questionnaire, of which twenty‐nine met the inclusion criteria. Healthy volunteers were younger and reported a higher EQ‐5D VAS score than the patient group (Table 1).

**TABLE 1:** Baseline characteristics

	MRgRT	Conventional EBRT	RARP	AS	Healthy volunteers
n	21	21	17	21	29
Age (median [IQR])	69 (67–73)	73 (67–75)	69 (67–71)	69 (63–73)	67 (63–71)
BMI (median [IQR])	24.9 (22.6–29.2)	26.0 (24.4–27.8)	25.8 (24.3–27.2)	24.7 (24.4–26.2)	24.5 (22.8–26.2)
Marital status					
Single	1 (4.8)	2 (9.5)	1 (5.9)	0 (0.0)	0 (0.0)
Living together	2 (9.5)	1 (4.8)	1 (5.9)	2 (9.5)	3 (10.3)
Married	16 (76.2)	17 (81.0)	14 (82.4)	18 (85.7)	24 (82.8)
Divorced	0 (0.0)	0 (0.0)	1 (5.9)	1 (4.8)	2 (6.9)
Widow	2 (9.5)	1 (4.8)	0 (0.0)	0 (0.0)	0 (0.0)
Education (%)					
No school completed	0 (0.0)	0 (0.0)	0 (0.0)	1 (4.8)	0 (0.0)
Primary school	1 (4.8)	0 (0.0)	1 (5.9)	1 (4.8)	0 (0.0)
High school	4 (19.0)	5 (23.8)	2 (11.8)	3 (14.3)	9 (31.0)
Vocational education	2 (9.5)	5 (23.8)	4 (23.5)	5 (23.8)	3 (10.3)
Higher education/university	14 (66.7)	11 (52.3)	10 (58.8)	11 (52.4)	17 (58.6)
Current employment (%)					
Full‐time	3 (14.3)	1 (4.8)	1 (5.9)	6 (28.6)	12 (41.4)
Part‐time	3 (14.3)	2 (9.5)	2 (11.8)	3 (14.3)	1 (3.4)
Unemployed/retired	15 (71.4)	18 (85.7)	14 (82.4)	12 (57.1)	16 (55.2)
Prostate cancer risk classification (EAU) (%)					
Low	1 (4.8)	2 (9.5)	1 (5.9)	17 (81.0)	NA
Intermediate	18 (85.7)	2 (9.5)	10 (58.8)	3 (14.3)	NA
High	2 (9.5)	17 (81.0)	6 (35.3)	1 (4.8)	NA
Androgen deprivation therapy (%)	3 (14.3)	15 (71.4)	0 (0.0)	0 (0.0)	NA
EQ‐5D‐5L dimensions (mean [SD])*					
Mobility	1.6 (1.0)	1.5 (0.8)	1.3 (0.6)	1.2 (0.5)	1.1 (0.3)
Self‐care	1.1 (0.4)	1.1 (0.2)	1.0 (0.0)	1.1 (0.5)	1.0 (0.0)
Daily activity	1.4 (0.7)	1.4 (0.9)	1.2 (0.4)	1.1 (0.4)	1.1 (0.4)
Pain/discomfort	1.6 (0.7)	2.0 (1.0)	1.9 (0.8)	1.6 (0.9)	1.6 (0.7)
Anxiety/depression	1.2 (0.4)	1.3 (0.7)	1.2 (0.5)	1.2 (0.4)	1.1 (0.4)
EQ‐5D VAS (median [IQR])	89 (75–91)	81 (70–90)	85 (80–90)	81 (80–90)	90 (81–97)

*Note*: All data available.

Abbreviations: AS, active surveillance; BMI, body mass index; EAU, European Association of Urology; EBRT, external beam radiotherapy; EQ‐5D‐5L, EuroQol 5‐dimension 5‐level questionnaire; IQR, interquartile range; MRgRT, magnetic resonance‐guided adaptive radiotherapy; NA, not applicable; SD, standard deviation; RARP, robot‐assisted radical prostatectomy; VAS, visual analogue scale.

**FIGURE 1:**
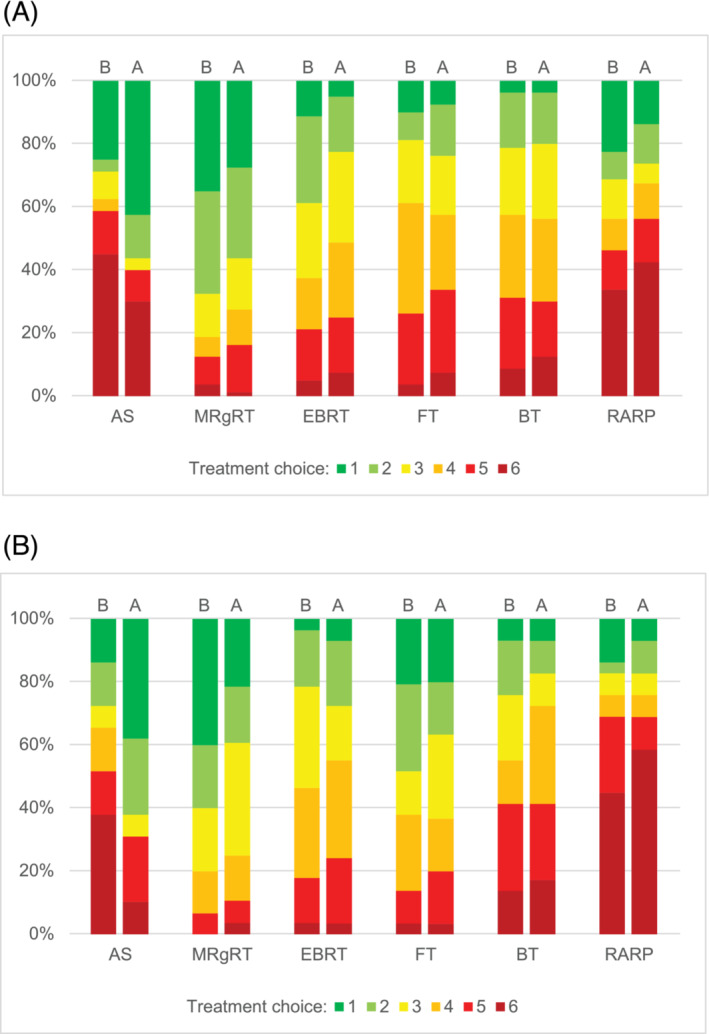
Preferences for treatment of localised prostate cancer before and after treatment information was received (first choice to sixth choice) for (A) patients (*n* = 80) and (B) healthy volunteers (*n* = 29). A, after information is received; B, before information is received; AS, active surveillance; EBRT, conventional external beam radiotherapy; FT, focal therapy; MRgRT, magnetic resonance‐guided adaptive radiotherapy; RARP, robot‐assisted radical prostatectomy

After receiving limited (one sentence) treatment‐outcome scenario information, patients ranked MRgRT most often as their first choice (33%), followed by AS (23%) and RARP (21%) (Figure 1). AS was most often ranked as the sixth choice (45%), followed by RARP (34%) and BT (9%). Patients ranked the treatment they had received themselves most often first, with 67% AS as first choice for AS patients, 86% MRgRT as first choice for MRgRT patients, 38% of conventional EBRT as first choice for conventional EBRT patients and 65% of RARP as first choice for RARP patients. Fifty‐five percent of patients that ranked AS as first choice, ranked RARP as sixth choice and 67% of participants that ranked RARP as first choice also ranked AS as sixth choice.

**FIGURE 2:**
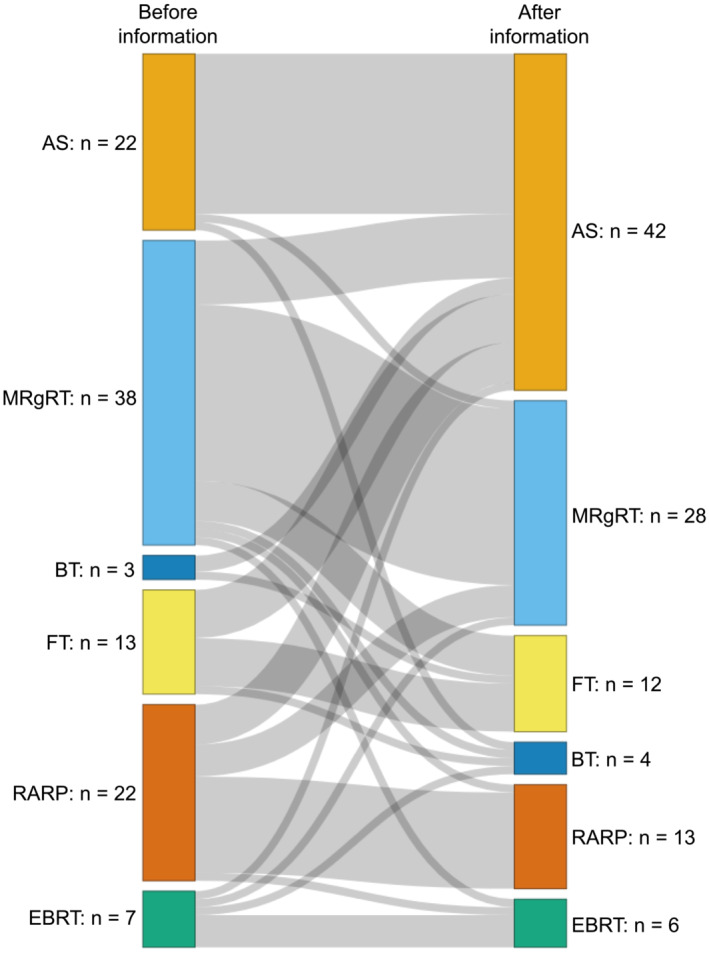
Mitigation of first‐choice treatment for localised prostate cancer after information was received of patients and healthy volunteers (*n* = 105). Four patients were excluded because multiple first choices were filled out. AS, active surveillance; EBRT, conventional external beam radiotherapy; FT, focal therapy; MRgRT, magnetic resonance‐guided adaptive radiotherapy; RARP, robot‐assisted radical prostatectomy

After receiving all the information from the different treatment‐outcome scenarios, the number of first choices increased for AS, remained the same for BT, and decreased for all other treatments (Figure 2). AS was most often ranked as first choice (43%), followed by MRgRT (28%) and RARP (14%). RARP was most often ranked as the sixth choice (42%), followed by AS (30%) and BT (12%). AS, MRgRT and RARP patients ranked the treatment they received themselves most often first with 86% AS as first choice for AS patients, 48% MRgRT as first choice for MRgRT patients and 41% RARP as first choice for RARP patients, which was an increase for AS and a decrease for MRgRT and RARP. Conventional EBRT patients most often ranked MRgRT as first choice (33%). Patients that ranked AS as first choice, most often ranked RARP as sixth choice (59%) and participants that ranked RARP as first choice most often ranked AS as sixth choice (55%).

Healthy volunteers ranked MRgRT most often as first choice (41%), followed by FT (21%), and both RARP and AS (14%) before receiving information. RARP (43%), AS (37%) and BT (13%) were most often ranked as sixth choice. After treatment information was received, AS was most often ranked as first choice (38%), followed by both MRgRT (21%) and FT (21%). RARP (61%), BT (18%) and AS (11%) were most often ranked as sixth choice.

VAS scores for the treatment‐outcome scenarios correlated strongly with TTO scores for both the patient group (*ρ* = 0.75, *p* < 0.001) and the healthy volunteer group (*ρ* = 0.73, *p* < 0.001). VAS scores were significantly different between the six treatment‐outcome scenarios and were highest for the AS treatment scenario (Table 2). After stratification for treatment history, age, education level and EQ‐5D VAS score, the VAS scores between the six treatment‐outcome scenarios remained significantly different, except for the treatment history group that was previously treated with RARP.

**TABLE 2:** Median score on the visual analogue scale for each treatment scenario of all participants and stratified by baseline characteristics

Participants	*n*	AS	MRgRT	Conventional EBRT	FT	BT	RARP	p^a^
Total	109	85 (70–93)	80 (69–90)	75 (60–85)^b^	80 (70–90)	72 (60–80)^b^	65 (54–80)^b^	<0.001
Patients	80	83 (70–92)	80 (69–90)	75 (60–83)^b^	80 (70–90)	72 (59–80)^b^	63 (50–80)^b^	<0.001
Healthy volunteers	29	85 (75–93)	75 (70–85)	75 (61–89)	80 (78–90)	72 (70–80)	65 (59–80)^b^	<0.001
Treatment								
AS	21	77 (75–90)	70 (61–80)^b^	61 (50–70)^b^	75 (66–85)	60 (50–80)^b^	50 (45–60)^b^	<0.001
MRgRT	21	81 (70–93)	90 (80–93)	75 (60–88)	72 (69–90)	80 (64–90)	60 (40–80)^b^	<0.001
Conventional EBRT	21	90 (70–94)	80 (70–90)	80 (70–90)	90 (80–95)	73 (70–90)	70 (60–80)	0.001
RARP	17	80 (62–90)	75 (58–82)	75 (65–80)	80 (75–85)	75 (60–80)	80 (75–85)	0.999
Age								
<70	58	85 (75–93)	80 (70–90)	74 (60–81)^b^	85 (77–90)	75 (60–80)^b^	60 (51–80)^b^	<0.001
≥70	51	81 (70–91)	80 (68–90)	79 (61–89)	79 (66–85)	70 (60–80)	70 (60–80)^b^	0.020
Education level^c^								
Low	46	78 (63–90)	75 (66–81)	71 (60–81)	80 (70–85)	70 (50–80)^b^	60 (55–80)^b^	<0.001
High	63	90 (75–94)	80 (70–90)	75 (64–89)^b^	85 (72–90)	76 (70–90)^b^	69 (53–80)^b^	<0.001
EQ‐5D VAS								
<80	31	80 (73–90)	80 (70–85)	70 (60–80)	80 (70–90)	73 (60–80)	65 (59–77)^b^	<0.001
≥80	78	85 (70–95)	79 (69–90)	75 (61–89)^b^	80 (71–90)	72 (60–84)^b^	63 (50–80)^b^	<0.001

*Note*: Visual analogue scale score of 0 is worst and 100 is best. All data available.

Abbreviations: AS = active surveillance; EBRT = external beam radiotherapy; FT = focal therapy; MRgRT = magnetic resonance‐guided adaptive radiotherapy; RARP = robot‐assisted radical prostatectomy; VAS = visual analogue scale.

^a^ Based on a Friedman test for repeated measures for indicating a significant difference in VAS score between all treatment scenarios.

^b^ Indicates a significant difference in VAS score between the AS scenario and the indicated treatment scenario based on a Wilcoxon signed rank test.

^c^ High education level includes higher education/university, and low education includes any other lower form of education.

TTO scores between the treatment‐outcome scenarios were significantly different for the total population and all strata except for the patients previously treated with RARP and patients of ≥70 years old (Supporting Information S2).

## 4 DISCUSSION

Preferences for treatment of localised PCa vary substantially between men and depend on the level and content of information received about the treatment procedures and outcomes. AS was the most preferred treatment choice after treatment information was received, followed by MRgRT, which suggests a preference towards no treatment or non‐invasive treatment in our study population. A preference towards AS and non‐ and minimal‐invasive alternatives becomes apparent from the reported VAS scores for the treatment‐outcome scenarios. Conventional EBRT, BT and RARP had significantly lower VAS scores as compared to AS, while MRgRT and FT had similar scores.

The main advantage of AS is that no radical treatment is used, so no treatment‐induced toxicity occurs. However, AS is generally only indicated for low‐risk PCa patients. Also, in the case of disease progression, radical treatment such as surgery or radiotherapy – with its sequelae – may be indicated.^20^ The psychological burden of living with untreated PCa may not be bearable for every patient, which may explain why AS was also reported to be the least preferred treatment option, before and after treatment information was received.^21^

MRgRT enables real‐time MR imaging and plan adaptation during radiotherapy, and therefore no fiducial markers need to be implanted.^22^ The treatment is completely non‐invasive and potentially causes less toxicity than conventional EBRT making it an appealing alternative to conventional EBRT.^23^ However, the reduction of toxicity is still hypothetical as clinical evidence is still lacking and long‐term evaluation is ongoing.^24^ Therefore, in the scenario descriptions, toxicity and survival outcomes for the MRgRT treatment‐outcome scenario were set to be identical to conventional EBRT. The current absence of evidence for lower toxicity may explain why several patients in our study preferred conventional EBRT over MRgRT.

FT is an experimental treatment. Literature reports low toxicity, but studies are limited by small samples and short follow‐up.^25^ Despite these uncertainties, the FT treatment‐outcome scenario VAS and TTO scores were among the highest. FT was the fourth most often selected as first‐choice treatment, before and after treatment information was received. This discrepancy may be influenced by the favourable toxicity outcomes on the one hand, but the uncertainties in terms of (biochemical free) survival and the need for re‐treatment on the other hand. Patients that consider FT may therefore eventually prefer a completely expectative approach without any treatment‐related toxicity, such as AS or radical treatment, with more certainty of having biochemical free survival.

BT was least often selected as the first choice, before and after treatment information was received. In the realm of radiotherapy, BT is the most invasive treatment. The risks of developing urinary and bowel complaints are marginally higher compared to conventional EBRT and MRgRT. Important advantages are the relatively short treatment in‐hospital duration and the relatively low erectile dysfunction rate after treatment. However, from our results, we can conclude that for most participants, the advantages of BT do not weigh up against the disadvantages.

RARP was ranked third most often as first choice before and after treatment information was received, indicating RARP to be a relatively preferred treatment. Contradictory, in the total study population, utility scores for RARP were among the lowest, which can be explained by the invasive treatment procedure and the relatively high rate of urinary incontinence and erectile dysfunction after treatment. However, the group that was previously treated with RARP did not report lower but similar utility scores for the RARP outcome‐scenario as for the other treatment scenarios, which suggests that there may be a distinct group that prefers RARP above the other less‐invasive (i.e. no catheter, no hospitalisation, and/or no incisions) treatment options.

Treatment preferences changed after information about the different treatment options was received. For example, AS was the first choice for 21% of participants before information was received and for 41% after information was received. For RARP, this was 19% versus 12%. The idea of having to eradicate the cancer by removing it from the body can be a logical first thought, explaining a relatively high preference for RARP and a low preference for AS in the first instance, as well as why patients that ranked RARP as first choice, most often ranked AS as sixth choice. Fear may lead to less rational decision‐making, especially when diagnosed with low‐grade cancer.^26^, ^27^ Our results suggest that AS is more accepted after extensive treatment information. Therefore, we advocate providing adequate information about treatment options, in particular for AS as an option for lower‐grade cancers.

Our study encourages future research and development into AS and non‐ and minimal‐invasive treatments such as MRgRT and FT. There is a demand for new technologies such as MRgRT and FT by patients and physicians, which also may have influenced the preference towards these treatments in this study.^28^ New technologies often promise favourable outcomes but may be costly. Early HTA may provide insight into the requirements of these innovations, in terms of costs and toxicity reduction, to be a cost‐effective alternative compared to standard treatments.^9^ The utility scores from this study can be used for early HTA.^10^, ^29^

This study has some limitations. Firstly, the majority of the men that filled out the questionnaire had previously been treated for localised PCa. In a previous paper, we described the first‐year PRO of the patient groups where the patients from the current study are part of.^30^ It indicates the toxicity that had been experienced by patients at the moment of filling out the current study's questionnaire. Both good and bad experiences with a previously received treatment may influence treatment preferences and utility scores.^19^ For example, most conventional EBRT and some MRgRT patients that participated in this study, received neoadjuvant androgen deprivation therapy (ADT). Their own treatment experience may have been negatively impacted by the ADT, especially with regard to erection problems, whereas all radiotherapy treatment‐outcome scenarios in our study were based on non‐ADT treatment. To minimise the influence of the patients' own treatment experience, we invited equal numbers of AS, MRgRT, conventional EBRT and RARP patients and included them at least six months after treatment as a washout period. We were not able to invite BT and FT patients as both treatments are not routinely performed at our institution. We also explicitly asked patients to assess the questionnaire not from their own PCa scenario but from the hypothetical PCa scenario that was described in the questionnaire. Furthermore, a reference group of healthy volunteers were invited by the participating patients who were asked to independently fill out the questionnaire. We found that the healthy volunteer group showed a similar pattern of preferences and utility scores compared to the patient group.

Secondly, for the healthy volunteer group that consisted of men older than 50 years, it should be noted that it is unknown what their initial knowledge of prostate cancer treatment was at the moment of filling out the questionnaire. The initial level of understanding of the one‐sentence treatment description for the six treatment options may vary between patients and even more so for the healthy volunteers participating in this study. This may have influenced their initial treatment ranking. However, PCa patients that are confronted with several treatment options at the time of diagnosis may have the same level of (scarce) knowledge.

Thirdly, the outcomes were based on literature and reviewed by a multidisciplinary team, aiming to represent the treatment procedures and outcomes as objectively as possible. Treatment‐outcome scenarios may, however, paint an optimistic or pessimistic picture of a certain treatment, which may differ from a patient's real‐life experience. Furthermore, patient's PCa risk classification and comorbidities, among other factors, may have an influence on treatment preferences and utility scores, and also treatment eligibility, which we did not account for in the present study. Moreover, for the toxicity profiles in the treatment‐outcome scenarios, we focused on the 1‐year outcomes as most toxicity for the different treatment options occurs during that time frame. However, some toxicities can occur after one year of follow‐up. For example, a minor but significant increase in gastrointestinal toxicity, predominantly bloody stools, after five years of follow‐up has been reported for radiotherapy treatment options (bloody stool about half the time or more frequently occurred in 1.3% for AS, 1.1% for radical prostatectomy and 5.6% for radiotherapy after five years of follow‐up [*p* < 0.001], as reported by Donovan et al.).^15^ For the aforementioned reasons, the advantages and disadvantages of a certain treatment may have been overestimated or underestimated by the participants, which therefore influenced treatment preference. Currently, we prospectively collect outcome data of all localised PCa patients treated in our region.^30^ The aim is to use these outcome data to update the treatment‐outcome scenario questionnaire for future studies on patient preferences for the treatment of localised PCa.

## 5 CONCLUSION

AS and non‐invasive treatment for localised PCa were most preferred by patients treated for localised prostate and healthy volunteers and received among the highest utility scores. Preference for the different treatments strongly depended on the level of information received: with more information about the procedure and outcomes, patients moved towards a preference for AS or non‐invasive treatment.

## DISCLOSURE OF INTEREST

The authors declare the following financial interests/personal relationships which may be considered as potential competing interests: HV receives research funding from Elekta. The remaining authors declare no potential competing interests.

## AUTHOR CONTRIBUTIONS

All authors have made substantial contributions to the material submitted for publication, all have read and approved the manuscript.

## Supporting information


**Data S1:** Survival and toxicity as used in the treatment‐outcome scenarios.

**Data S2:** Median time trade‐off score for each treatment scenario of all participants and stratified by baseline characteristics

**Data S3:** Treatment‐outcome scenario questionnaire translated from the original Dutch questionnaire
