# Supplementary material for: Patient preferences for treatment modalities for localised prostate cancer
Source: BJUI Compass. 2022 Nov 17;4(2):214–22. doi: 10.1002/bco2.198 (PMC9931535; doi:10.1002/bco2.198)
Supplement: Supplementary file 1 — Data S1: Survival and toxicity as used in the treatment‐outcome scenarios. [file BCO2-4-214-s002.docx]

**Supplement 1:**

Survival and toxicity as used in the treatment-outcome scenarios.

|  |  |  | New-onset complaints 1 year after treatment: | | | | | |
| --- | --- | --- | --- | --- | --- | --- | --- | --- |
| Treatment-outcome scenario | 10-year mortality | Chance of PCa recurrence | Unwanted urine loss | use of continence aids | Total incontinence | Urinairy urgency | Diarrhea | Erectile dysfunction |
| AS^a^ | 1% | NA | 7% | 2% | 1% | 24% | 9% | 16% |
| MRgRT | 1% | 10% | 23% | 11% | 2% | 48% | 15% | 56% |
| Conventional EBRT | 1% | 10% | 23% | 11% | 2% | 48% | 15% | 56% |
| FT^b^ | 1% | > 10% | 7% | 1% | < 1% | 7% | < 1% | 24% |
| BT | 1% | 10% | 26% | 14% | 2% | 65% | 24% | 28% |
| RARP | 1% | 10% | 60% | 54% | 4% | 29% | < 4% | 76% |

Abbreviations: AS = active surveillance; MRgRT = Magnetic resonance guided adaptive radiotherapy; EBRT = external beam radiotherapy; FT = focal therapy; RARP = robot-assisted radical prostatectomy; PCa = prostate cancer; NA = not applicable

^a^ In the AS treatment-outcome scenario, explained is that toxicity as described is caused by natural aging. It is also explained that AS patients are regularly been followed up and in case of tumor progression, radical treatment may be indicated and toxicity as described for the radical treatment options may occur. In 10 years, approximately 50% of AS patients undergo radical treatment.

^b^ In the FT treatment-outcome scenario, explained is that FT is still in its experimental phase and therefore exact percentages of recurrence, mortality, and toxicity are unknown and possibly higher that reported. Also, it is explained that the chance of recurrence is probably higher than after radical therapy. In case of recurrence, radical treatment may be indicated and toxicity as described for the radical treatment options may occur.
