# Supplementary material for: Patient preferences for treatment modalities for localised prostate cancer
Source: BJUI Compass. 2022 Nov 17;4(2):214–22. doi: 10.1002/bco2.198 (PMC9931535; doi:10.1002/bco2.198)
Supplement: Supplementary file 2 — Data S2: Median time trade‐off score for each treatment scenario of all participants and stratified by baseline characteristics [file BCO2-4-214-s003.docx]

**Supplement 2:**

Median time trade-off score for each treatment scenario of all participants and stratified by baseline characteristics

|  | n | AS | MRgRT | Conventional EBRT | FT | BT | RARP | p^a^ |
| --- | --- | --- | --- | --- | --- | --- | --- | --- |
| Total | 109 | 8.0 (6.7-9.3) | 8.0 (7.0-9.0) | 8.0 (6.9-9.1) | 8.1 (7.0-9.5) | 8.0 (6.5-9.0) | 7.0 (5.9-8.5)^b^ | < 0.001 |
| Patients | 80 | 8.1 (6.5-9.5) | 8.0 (7.0-9.3) | 8.0 (6.9-9.2) | 8.0 (7.0-9.5) | 8.0 (6.5-9.0) | 7.0 (6.0-8.5)^b^ | < 0.001 |
| Healthy volunteers | 29 | 8.0 (7.0-9.0) | 7.6 (7.0-8.6) | 8.0 (6.9-9.0) | 8.8 (7.3-9.0) | 8.0 (7.0-8.9) | 7.0 (5.4-8.0)^b^ | < 0.001 |
| Treatment | | | | | | | | |
| AS | 21 | 8.0 (6.5-9.5) | 7.5 (6.7-8.0) | 7.0 (5.4-8.0) | 8.0 (7.0-9.0) | 7.0 (5.0-8.4) | 6.5 (5.8-8.0) | 0.011 |
| MRgRT | 21 | 8.0 (7.0-9.0) | 9.0 (7.6-9.4) | 8.0 (7.0-9.0) | 8.0 (6.6-9.0) | 8.4 (7.5-9.0) | 7.0 (6.0-8.4) | < 0.001 |
| Conventional EBRT | 21 | 9.0 (6.0-9.8) | 8.3 (7.6-9.7) | 9.2 (8.0-9.9) | 9.1 (8.0-9.8) | 8.7 (7.5-9.3) | 7.3 (6.1-8.8) | 0.002 |
| RARP | 17 | 8.0 (6.3-9.3) | 7.2 (6.0-8.7) | 7.2 (7.0-9.0) | 7.6 (7.1-9.5) | 8.0 (6.8-9.0) | 8.1 (7.0-9.0) | 0.999 |
| Age | | | | | | | | |
| < 70 | 58 | 8.8 (7.2-9.5) | 8.3 (7.0-9.3) | 8.0 (7.0-9.0) | 9.0 (7.7-9.5) | 8.0 (6.9-9.0) | 7.0 (5.9-8.5)^b^ | < 0.001 |
| ≥ 70 | 51 | 7.5 (6.0-9.3) | 7.6 (6.6-9.0) | 8.0 (6.9-9.3) | 7.6 (6.3-9.0) | 8.0 (6.4-8.9) | 7.3 (5.8-8.4) | 0.443 |
| Education level^c^ | | | | | | | | |
| Low | 46 | 7.5 (6.3-9.0) | 7.5 (6.0-8.5) | 7.5 (6.8-8.4) | 8.0 (6.4-9.0) | 7.5 (6.1-8.5) | 7.0 (6.0-8.0) | 0.010 |
| High | 63 | 9.0 (7.1-9.6) | 8.4 (7.2-9.4) | 8.0 (7.0-9.5) | 8.5 (7.1-9.5) | 8.5 (7.0-9.0) | 7.3 (5.7-8.6)^b^ | < 0.001 |
| EQ-5D VAS | | | | | | | | |
| < 80 | 31 | 7.6 (6.2-9.0) | 8.0 (7.1-8.6) | 8.0 (5.8-8.4) | 8.0 (6.5-9.5) | 8.0 (5.6-9.0) | 7.0 (6.0-8.0) | 0.010 |
| ≥ 80 | 78 | 8.3 (7.0-9.5) | 8.0 (7.0-9.4) | 8.0 (7.0-9.3) | 8.7 (7.0-9.5) | 8.0 (6.9-9.0) | 7.4 (5.8-8.5)^b^ | < 0.001 |

AS = active surveillance; MRgRT = Magnetic resonance guided adaptive radiotherapy; EBRT = external beam radiotherapy; FT = focal therapy; RARP = robot-assisted radical prostatectomy; VAS = visual analog scale.

^a^ Based on a Friedman test for repeated measures for indicating a significant difference in VAS score between all treatment scenarios.

^b^ Indicates a significant difference in VAS score between the AS scenario and the indicated treatment scenario based on a Wilcoxon signed rank test.

^c^ High education level includes higher education/university, and low education includes any other lower form of education.
