# Supplementary material for: Patient preferences for treatment modalities for localised prostate cancer
Source: BJUI Compass. 2022 Nov 17;4(2):214–22. doi: 10.1002/bco2.198 (PMC9931535; doi:10.1002/bco2.198)
Supplement: Supplementary file 3 — Data S3: Treatment‐outcome scenario questionnaire translated from the original Dutch questionnaire [file BCO2-4-214-s001.pdf]

Supplement 3:

Treatment-outcome scenario questionnaire translated from the original Dutch questionnaire

Introduction

With this questionnaire we want to gain insight into the treatment preferences of patients with prostate cancer. It does not matter if you have or have had prostate cancer and how you have been treated for prostate cancer.

Filling out this questionnaire is anonymous, the person who sent you the questionnaire will not have access to your personal answers. Please complete the questionnaire according to your own opinion and try not to be influenced by others. The questionnaire starts with some general questions. This is followed by questions about your health and well-being, activities and work. You will then be asked a number of questions about the treatment of prostate cancer.

General questionnaire

The questionnaire starts with some general questions. This is followed by questions about your health and well-being, activities and work.

What is your age? ___________ years.

What is your sex?
☐ male
☐ female

What is your marital status?
☐Single
☐Living with partner
☐Married/registered partnership
☐Divorced
☐Widower

What is the highest level of education you have completed?
☐ No education completed
☐ Primary education (primary school)
☐ Pre-vocational secondary education (VMBO/LBO/MAVO)
☐ Higher Pre-vocational secondary education (HAVO)/preparatory scientific education (VWO)
☐ Secondary vocational education (MBO)
☐ Higher vocational education (HBO)
☐ University

What is your current work situation?
☐ I work fulltime – skip next question
☐ I work parttime – skip next question
☐ I do not work

What is the reason that you do not work?
☐ I am retired
☐ I am unemployed
☐ I am incapacitated for work
☐ I am a housekeeper

What is your hight? ____________ cm
If you don't know exactly, give an estimate.

What is your weight? ____________ kg (please round to whole kilograms)
If you don't know exactly, give an estimate.

Do you smoke regularly (almost every day)?
☐ Yes
☐ No, I quit smoking in ____________ / when I was ____________ years old
☐ Never smoked
At what age did you start smoking?____________ Years

Do you have or have had prostate cancer?
☐ Yes
☐ No -> end of questionnaire

Have you ever been treated for prostate cancer?

☐ Yes	
☐ No	
☐ No, only active surveillance	Date start active surveillance:
If yes:	
☐ External radiotherapy	Date start treatment:
☐ Surgical prostate removal	Date surgery:
☐ Internal radiotherapy (brachytherapy)	Date treatment:
☐ Otherwise:_____________________________________	Date treatment:

EuroQol questionnaire (EQ-5D-5L)

Under each heading, please tick the ONE box that best describes your health TODAY.

MOBILITY	
I have no problems in walking about	☐
I have slight problems in walking about	☐
I have moderate problems in walking about	☐
I have severe problems in walking about	☐
I am unable to walk about	☐

SELF-CARE	
I have no problems washing or dressing myself	☐
I have slight problems washing or dressing myself	☐
I have moderate problems washing or dressing myself	☐
I have severe problems washing or dressing myself	☐
I am unable to wash or dress myself	☐

USUAL ACTIVITIES (e.g. work, study, housework, family or leisure activities)	
I have no problems doing my usual activities	☐
I have slight problems doing my usual activities	☐
I have moderate problems doing my usual activities	☐
I have severe problems doing my usual activities	☐
I am unable to do my usual activities	☐

PAIN / DISCOMFORT	
I have no pain or discomfort	☐
I have slight pain or discomfort	☐
I have moderate pain or discomfort	☐
I have severe pain or discomfort	☐
I have extreme pain or discomfort	☐

ANXIETY / DEPRESSION	
I am not anxious or depressed	☐
I am slightly anxious or depressed	☐
I am moderately anxious or depressed	☐
I am severely anxious or depressed	☐
I am extremely anxious or depressed	☐

- We would like to know how good or bad your health is TODAY.
- This scale is numbered from 0 to 100.
- 100 means the best health you can imagine.
  0 means the worst health you can imagine.
- Mark an X on the scale to indicate how your health is TODAY.
- Now, please write the number you marked on the scale in the box below

YOUR HEALTH TODAY = [ ]

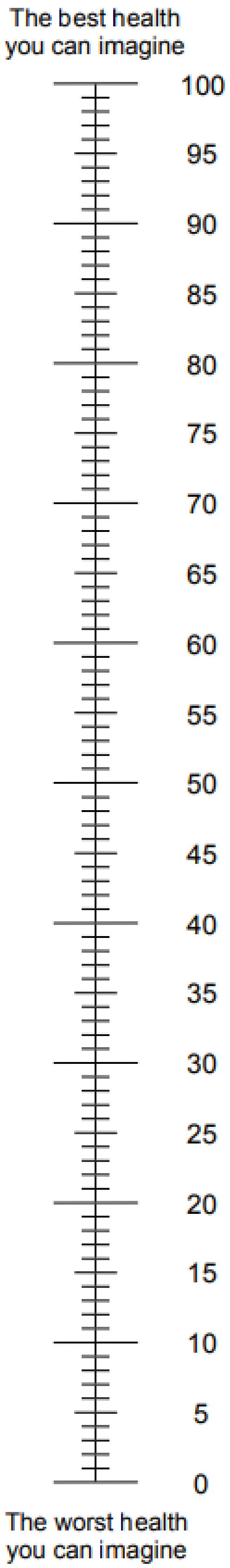

Questionnaire treatment preference prostate cancer

When completing the following question, it is important that you do not look at it from your own situation, but from the situation described below:

Diagnosis:

You have been told that you have prostate cancer and that you need to undergo treatment. You have not been treated for prostate cancer before. The prostate cancer was discovered after blood tests in which the PSA (prostate specific antigen) was found to be elevated. Subsequently, biopsies (small bites of prostate tissue) were taken from the prostate in which the pathologist found prostate cancer. The tumor is well treatable because the tumor tissue is only within the prostate and has not spread, so the chance that you will die from prostate cancer is very small. However, there are various treatment options for which you are eligible, each with its advantages and disadvantages.

Please indicate in the next question which treatment for prostate cancer you would prefer in first instance, assuming that you are eligible for all 6 treatments. Your first impression counts, before we give you information about each treatment. After this first question, each treatment is explained in detail.

You can indicate your 1st to 6th choice. You may only use each number once.

…… Scenario 1: Active surveillance of the prostate cancer

…… Scenario 2: Surgical removal of the prostate using the surgical robot (Da Vinci)

…… Scenario 3: External radiotherapy with placing 4 gold markers (1 x 5 millimeters) in the prostate for position determination

…… Scenario 4: MR-Linac guided external radiotherapy with support of MRI scans for position determination during the entire radiotherapy (without placing gold markers in the prostate)

…… Scenario 5: Internal radiotherapy by placing small radioactive seeds in the prostate (LDR "low dose rate" brachytherapy)

…… Scenario 6: Focal therapy in which only the tumor in the prostate is treated via a number of needles and not the entire prostate (Irreversible Electroporation/Nanoknife)

Don't try to go back to rearrange the order. Your fist impression counts. At the end of this questionnaire, you will be asked the same question again, after you have been informed about each treatment method.

Treatment-outcome scenarios

We would like to outline a number of scenarios for the treatment of prostate cancer. Each scenario describes a different treatment. The different treatments have different sequelae, such as different side effects and differences in recovery. The chance of the disease returning also differs. The chance of dying from the disease is not different for the different treatments.

In the scenarios about prostate cancer, we assume a case of a patient with a type of prostate cancer who is eligible for all the described treatments. When answering the questions in the scenarios, it is important that you view it from the situation as described in the scenario and not from your own situation.

Please note: if you have been treated for prostate cancer, you may not have been eligible for all of the treatments described here. It is also possible that the side effects do not correspond with how you were informed about the side effects or how you experienced the side effects yourself. This is because every patient and every prostate cancer is different. Therefore, not all patients are eligible for all types of treatment and side effects can vary substantially from patient to patient. Also, some treatments are experimental and are not yet standard care. At the time of your diagnosis, there has been extensive consultation about which treatment you were eligible for, and those treatments with the side effects applicable to you were discussed with you. No treatments have ever been withheld from you.

We start with 2 example scenarios with explanations, then 2 practice scenarios and then 6 scenarios about prostate cancer treatment. The 2 example scenarios and 2 exercise scenarios are not yet about prostate cancer.

Example scenarios

1. You give a score: You give each scenario a score between 0 and 100. A score of 100 means the best health and a score of 0 means the worst health imaginable. Most people consider death to be the lowest possible score.

2. You express the time: For each scenario, imagine that you have a maximum of 10 years to live after you have received the treatment. You can choose whether you would like to exchange a number of months or years of these 10 years for life in perfect health (without the cancer treatment). We therefore ask you to consider for all scenarios how many months or years in your opinion are equal to the 10 years of life after treatment as described in the scenario.

Because these are difficult questions, we explain this with the help of two examples:

Example 1

You are involved in a serious car accident. You will suffer a spinal cord injury (paralysis), as a result you will end up in a wheelchair.

1. Score

On a scale of 0 to 100, what score would you give this scenario?

You rate this scenario with 40 points. You enter this as follows:

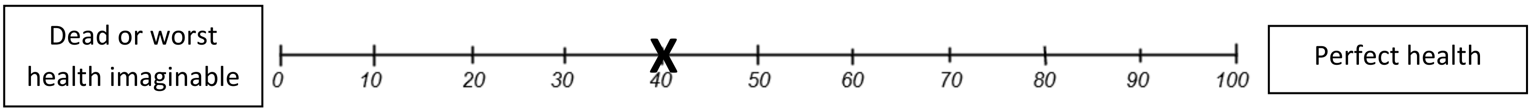

40 ...... points

2. Time

You will live another 10 years after the accident. In your opinion, how many years or months in full health is equivalent to living with a spinal cord injury for 10 years?

You believe that living 6 years in full health is equivalent to living 10 years after the described scenario (being wheelchair dependent).

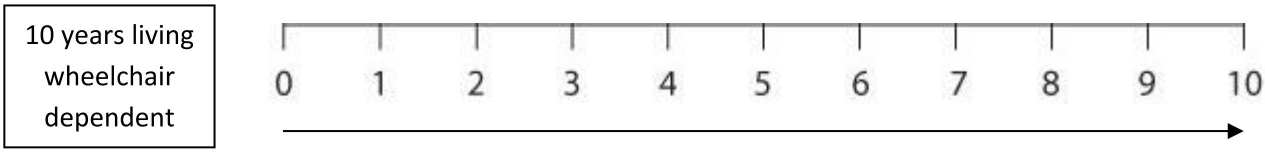

6 years of perfect health

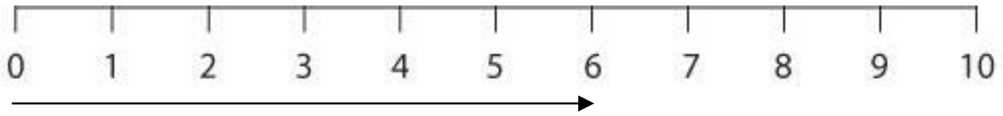

You enter the information as follows:

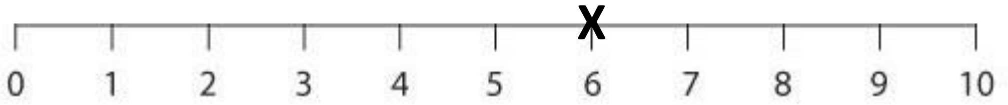

6 …… years

0 …… months

Example 2

You have hay fever that often makes you sneeze, have a runny nose and suffer from itchy and red eyes. This is especially the case in the spring and summer. Medication partially helps.

1. Score

On a scale of 0 to 100, what score would you give this scenario?

You rate this scenario with 90 points. You enter this as follows:

Dead or worst health imaginable

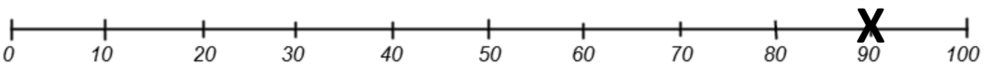

Perfect health

90 …… points

2. Time

You will live another 10 years with hay fever. In your opinion, how many years or months in full health is equivalent to living with hay fever for 10 years?

You are of the opinion that you would like to give up 3 months of the 10 years that you are still alive. So you think that 9 years and 9 months without hay fever is equal to 10 years with hay fever.

10 years of living with hay fever

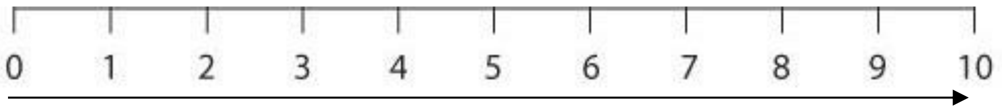

9 years and 9 months in perfect health

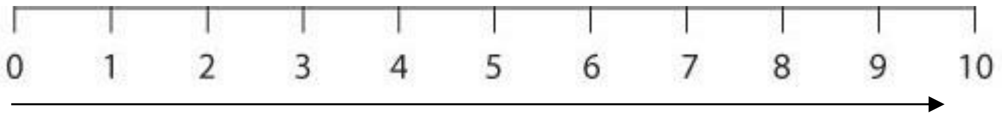

You enter the information as follows:

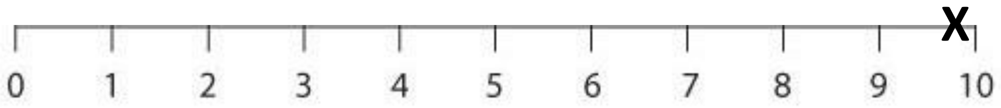

9 …… years

9 …… months

Practice scenarios

How you assess the scenarios is of course up to you. Previous examples are suggestions. Everyone has a different opinion. The aim of this survey is that you fill out your own opinion. The exercise scenarios are not yet about prostate cancer. We ask you to assess the following 2 scenarios as an exercise:

Practice scenario 1:

Paralysis after stroke

A stroke paralyzes the right side of your body. You also have problems with speaking due to the stroke and you have difficulty with reading and writing.

1. Score

On a scale of 0 to 100, what score would you give this scenario?

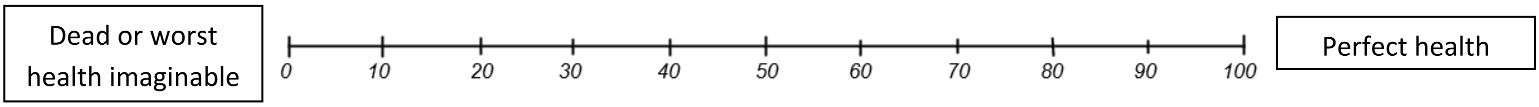

…… points

2. Time

How many years in perfect health, in your opinion, are equivalent to 10 years of life with the above scenario?

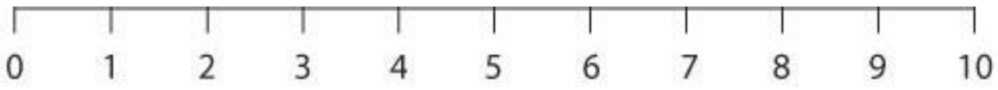

…… years

…… months

Practice scenario 2:

Wound on finger

You cut yourself in your finger while cooking. The doctor stitches the wound and after the wound has healed a small scar remains.

1. Score

On a scale of 0 to 100, what score would you give this scenario?

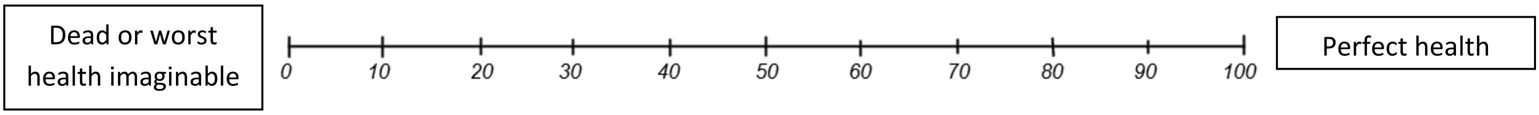

…… points

2. Time

How many years in perfect health, in your opinion, are equivalent to 10 years of life with the above scenario?

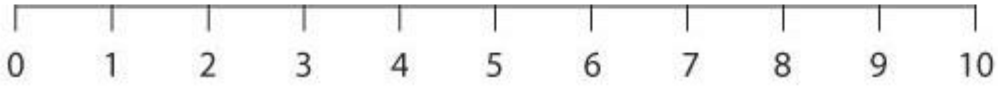

…… years

…… months

Prostate cancer treatment-outcome scenarios

When completing the following scenarios, it is important that you do not fill out the questions below based on your own situation, but based on the situation described below:

Diagnosis:

You have been told that you have prostate cancer and that you need to undergo treatment. You have not been treated for prostate cancer before. The prostate cancer was discovered after blood tests in which the PSA (prostate specific antigen) was found to be elevated. Subsequently, biopsies (small bites of prostate tissue) were taken from the prostate in which the pathologist found prostate cancer. The tumor is well treatable because the tumor tissue is only within the prostate and has not spread, so the chance that you will die from prostate cancer is very small. However, there are various treatment options for which you are eligible, each with its advantages and disadvantages.

We ask you to first read all 6 scenarios below. You can then answer the 2 questions below for each scenario.

Scenario 1:

Surgical removal of the prostate using a surgical robot (Da Vinci)

Treatment

You will undergo a surgical procedure in which the entire prostate and therefore the entire tumor is removed. The operation is performed using a surgical robot (Da Vinci), through 5 small openings in the abdomen (keyhole surgery). The procedure will take place under general anaesthetic. During the operation, a urinary catheter will be placed, through which you will urinate.

Recovery

You will be left with 5 small wounds of about 1.5 cm from the procedure, one of which is slightly larger because the prostate has been removed. The urinary catheter will need to be left in place for about 10 days. You can go home the day after surgery. The day before you go home, the nurse will teach you how to care for the urinary catheter at home. More than a week later, the urinary catheter is removed in the hospital. After the urinary catheter has been removed, you will be given incontinence material to take home. During the first 6 weeks you are not allowed to do heavy physical work, lift, cycle or play sports.

After the operation, the pelvic floor has to get used to the new situation and the sphincter of the bladder has to be trained. In the first time after the operation, many patients suffer from unwanted urine loss. However, it also happens that patients are (almost) 'dry' immediately after removal of the catheter. Most men are continent again 3 to 6 months after the operation, but some continue to have urine leakage afterwards.

Follow-up

You will come for follow-up 6 weeks after surgery, then at 3, 6, and 12 months, then every six months for up to 3 years, and annually for 5 to 10 years. During these visits, it will be checked whether your PSA (prostate specific antigen) does not change and whether you remain cancer free.

Prognosis

The chance that the prostate cancer will return within 10 years is about 10%

The chance that you will die from prostate cancer within 10 years is about 1%.

Adverse effects

The numbers below are for men without the specific complaints before the treatment. In men who already had one or more of the specific complaints before treatment, the complaints persisted or got worse.

- Unwanted urine leakage: approximately 60% of men experience unwanted urine leakage a year after surgery. 54% use incontinence material.
- Total incontinence: about 4% of men are totally incontinent one year after surgery.
- Increased urge to urinate: about 29% of men experience an increased urge to urinate one year after surgery.

• Diarrhea: less than 4% of men suffer from diarrhea one year after surgery.
• Erection problems: about 76% of men have erection problems a year after surgery.

1. Score

On a scale of 0 to 100, what score would you give this scenario?

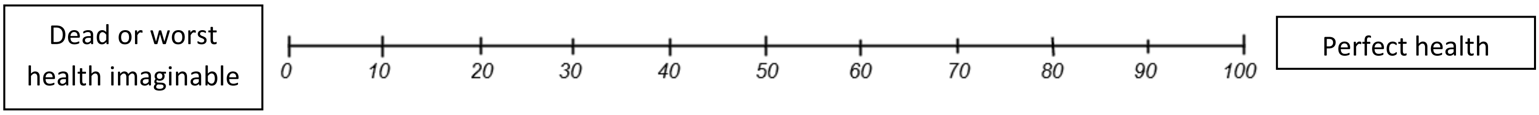

…… points

2. Time

How many years in perfect health, in your opinion, are equivalent to 10 years of life with the scenario above?

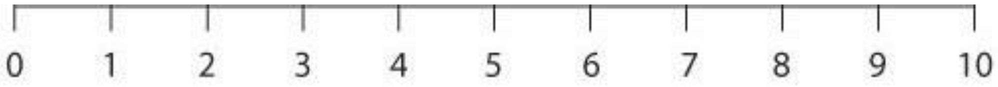

…… years

…… months

Scenario 2:

External radiotherapy with placing gold markers in the prostate

Treatment

You are undergoing treatment in which the prostate is irradiated from the outside. To properly visualize the prostate, an MRI scan and a CT scan will be made before radiation is applied. Also, by means of 2 punctures in the perineum (the skin between the scrotum and the anus) 4 gold markers of 1 by 5 millimeters will be placed in the prostate. Inserting the gold markers is similar to the biopsies (taking bites of prostate tissue) that the urologist performs to diagnose the prostate cancer. The gold markers are needed to properly visualize the position of the prostate prior to each irradiation.

You will be irradiated 5 times, spread over 2 and a half weeks. You are in the hospital for twenty minutes for each treatment. Before each irradiation, an X-ray will be taken on the irradiation machine to determine the position of the gold markers in the prostate so that you can be irradiated as accurately as possible.

Recovery

You will not feel anything from the irradiation itself. The body reacts with some delay to the radiation. Shortly after the radiation treatment, complaints may appear, after a month these complaints have usually subsided. It can be fatigue, irritation when urinating and/or intestinal complaints. There is a chance that the complaints will be permanent.

Follow-up

You will come for follow-up 6 weeks after treatment and then after 3, 6, and 12 months, then every six months for up to 3 years and annually for 5 to 10 years. In general, the follow-up takes place alternately with your referring doctor (urologist) and with the radiation oncologist. They keep each other informed. During these visits, it will be checked whether your PSA (prostate specific antigen) does not change and whether you remain cancer free.

Prognosis

The chance that the cancer will return within 10 years is about 10%

The chance that you will die from prostate cancer within 10 years is about 1%.

Adverse effects

The figures below are for men without the specific complaints before the treatment. In men who already had one or more of the specific complaints before treatment, the complaints persisted or got worse.

• Unwanted urine leakage: approximately 23% of men experience unwanted urine leakage a year after treatment. 11% use incontinence material.
• Total incontinence: about 2% of men are totally incontinent one year after treatment.

• Increased urge to urinate: about 48% of men experience an increased urge to urinate one year after treatment.
• Diarrhea: about 15% of men suffer from diarrhea a year after treatment.
• Erection problems: about 56% of men have erection problems a year after treatment.

1. Score

On a scale of 0 to 100, what score would you give this scenario?

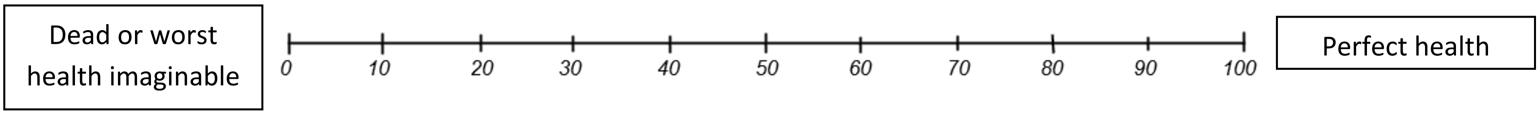

…… points

2. Time

How many years in perfect health, in your opinion, are equivalent to 10 years of life with the scenario above?

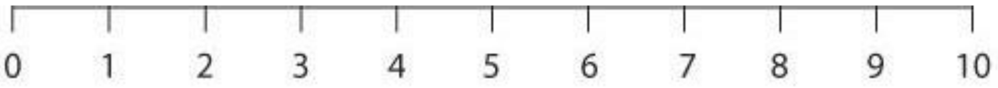

…… years

…… months

Scenario 3:

External radiotherapy with support from MRI scans during radiation (MR-Linac)

Treatment

You are undergoing treatment in which the prostate is irradiated from the outside. In order to properly visualize the prostate, an MRI scan will be made before radiation is applied.

You will be irradiated 5 times, spread over 2 and a half weeks. Prior to each irradiation, an MRI scan will be made in the position in which you are being irradiated to adjust the radiation to the position of the prostate. During the radiation treatment, MRI scans will be made on the device with which you are being irradiated to see whether the prostate is being properly targeted. You are in the hospital for one hour for each treatment.

Recovery

You will not feel anything from the irradiation itself. The body reacts with some delay to the radiation. Shortly after the radiation treatment, complaints may appear, after a month these complaints have usually subsided. It can be fatigue, irritation when urinating and/or intestinal complaints. There is a chance that the complaints will be permanent.

Follow-up

You will come for follow-up 6 weeks after treatment and then after 3, 6, and 12 months, then every six months for up to 3 years and annually for 5 to 10 years. In general, the follow-up takes place alternately with your referring doctor (urologist) and with the radiation oncologist. They keep each other informed. During these visits, it will be checked whether your PSA (prostate specific antigen) does not change and whether you remain cancer free.

Prognosis

The chance that the cancer will return within 10 years is about 10%

The chance that you will die from prostate cancer within 10 years is about 1%.

Adverse effects

Radiation with the support of MRI scans is a relatively new treatment. At this point, there is enough scientific evidence to suggest that the side effects are comparable to radiation with gold markers placed in the prostate (Scenario 2).

The figures below are for men without the specific complaints before the treatment. In men who already had one or more of the specific complaints before treatment, the complaints persisted or got worse.

• Unwanted urine leakage: approximately 23% of men experience unwanted urine leakage a year after treatment. 11% use incontinence material.
• Total incontinence: about 2% of men are totally incontinent one year after treatment.
• Increased urge to urinate: about 48% of men experience an increased urge to urinate one year after treatment.

• Diarrhea: about 15% of men suffer from diarrhea a year after treatment.
• Erection problems: about 56% of men have erection problems a year after treatment.

1. Score

On a scale of 0 to 100, what score would you give this scenario?

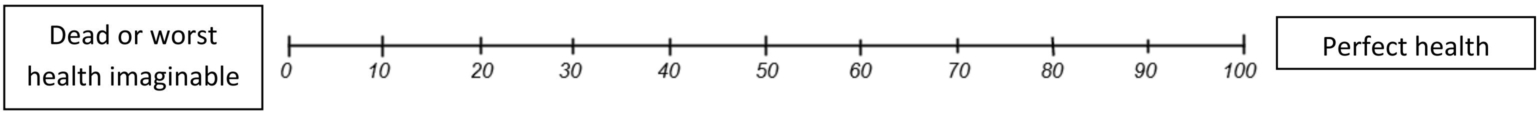

…… points

2. Time

How many years in perfect health, in your opinion, are equivalent to 10 years of life with the scenario above?

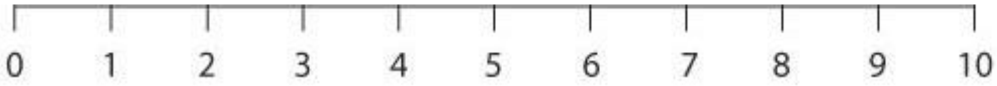

…… years

…… months

Scenario 4:

Internal radiotherapy by placing radioactive seeds in the prostate (LDR "low dose rate" brachytherapy)

Treatment

The radiation is done from within. To do this, the doctor places radioactive material in your prostate. This radioactive material consists of small metal seeds. The implantation of the radioactive seeds takes about two hours.

Before the treatment, the anesthesiologist will give you an anesthetic via an epidural injection. Once the anesthetic has taken effect, you will first be given a urinary catheter. The radiation oncologist inserts a number of very thin hollow needles into your prostate. This is done with the help of ultrasound images. These needles go through the perineum (the tissue between the scrotum and anus). It is then calculated how many seeds are needed and these seeds are prepared for implantation in the prostate. An average of 60 seeds are implanted, depending on the size of the prostate. After 6 months, the radioactivity of the seeds is negligible. These seeds remain in your prostate for the rest of your life.

Recovery

When most of the anesthesia has worn off, the urinary catheter is removed. When you can urinate on your own again, you can go home the same day. It is recommended not to do any heavy work for the first few days after the procedure and not to cycle for the first month. You can continue with all normal activities.

Follow-up

The first appointment is with the radiation oncologist, one month after the implantation. An MRI scan is also performed. You will then come for follow-up 3, 6, and 12 months after treatment, then every six months for up to 3 years and annually for 5 to 10 years. In general, the follow-up take place alternately with your referring doctor (urologist) and with the radiation oncologist. They keep each other informed. During these visits, it will be checked whether your PSA (prostate specific antigen) does not change and whether the cancer remains away.

Prognosis

The chance that the cancer will return within 10 years is about 10%.

The chance that you will die from prostate cancer within 10 years is about 1%.

Adverse effects

The figures below are for men without the specific complaints before the treatment. In men who already had one or more of the specific complaints before treatment, the complaints persisted or got worse.

• Unwanted urine leakage: approximately 26% of men have unwanted urine leakage a year after treatment. 14% use incontinence material.

• Total incontinence: about 2% of men are totally incontinent one year after treatment.
• Increased urge to urinate: about 65% of men experience an increased urge to urinate one year after treatment.
• Diarrhea: about 24% of men suffer from diarrhea one year after treatment.
• Erection problems: about 28% of men have erection problems a year after treatment.

1. Score

On a scale of 0 to 100, what score would you give this scenario?

Dead or worst health imaginable — 0 10 20 30 40 50 60 70 80 90 100 — Perfect health

…… points

2. Time

How many years in perfect health, in your opinion, are equivalent to 10 years of life with the scenario above?

0 1 2 3 4 5 6 7 8 9 10

…… years

…… months

Scenario 5:

Active surveillance of prostate cancer

Treatment

No so-called “radical” treatment is performed, but the prostate is closely monitored. We can do that because we know that prostate cancer grows very slowly.

Patients are given a physical examination and a blood test every three months, especially monitoring the PSA. A scan of the tumor is also made every year. Usually this is an MRI scan, but it is also possible that biopsies of the prostate are performed every year.

Follow-up

After 10 years, about half of the men will eventually have switched from active monitoring to radical treatment. In most cases this will be radiation or surgery (scenarios 1 to 4). Sometimes this is the wish of the patient, because he finds it an unpleasant idea that the prostate cancer is still present in the body, but in most cases it is because the tumor grows too much in the long term.

Prognosis

The chance that you will die from prostate cancer within 10 years is about 1%.

After 10 years, approximately 50% of patients have undergone surgical removal, or external or internal radiotherapy of the prostate, with the corresponding risk of side effects as described in scenarios 1 to 4.

Adverse effects

Because no invasive treatment is performed, there are no treatment-related side effects. However, there are complaints that fit with the natural course of aging, whether or not in combination with prostate cancer.

The figures below are for men without the specific complaints before the treatment. In men who already had one or more of the specific complaints before treatment, the complaints persisted or got worse.

- Unwanted urine leakage: approximately 7% of the men have unwanted urine leakage a year after the start of active monitoring. 2% use incontinence material.
- Total incontinence: about 1% of men are totally incontinent one year after the start of active surveillance.
- Increased urge to urinate: about 24% of men experience an increased urge to urinate one year after starting active surveillance.
- Diarrhea: about 9% of the men suffer from diarrhea one year after the start of active surveillance.
- Erection problems: about 16% of men have erection problems a year after starting surveillance.

1. Score

On a scale of 0 to 100, what score would you give this scenario?

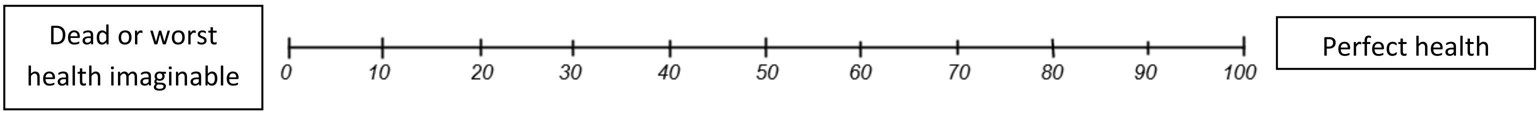

…… points

2. Time

How many years in perfect health, in your opinion, are equivalent to 10 years of life with the scenario above?

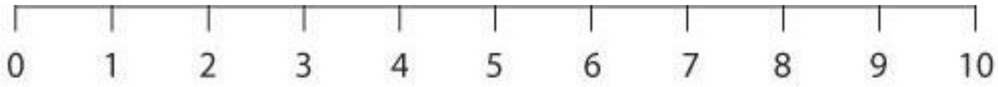

…… years

…… months

Scenario 6:

Focal therapy of prostate cancer (Irreversible Electroporation/Nanoknife)

Treatment

With a focal treatment, only the visible tumor (on ultrasound and MRI) in the prostate is treated and the rest of the prostate is treated as little as possible. The treatment is performed under general anesthesia. The urologist makes an ultrasound of the prostate and inserts a number of needles through the area between the anus and the scrotum. How many needles are needed depends on the size of the prostate tumor. Then a plan is calculated how much current should be given by each needle pair. This process destroys the cancer cells in the prostate. During the treatment, a urinary catheter is placed. You wake up with the urinary catheter. When the urologist has finished the treatment, he removes the needles and places a strong bandage on the place where the needles were placed.

Recovery

The urinary catheter will remain in place for approximately 3 days. The nurse will discuss with you how to care for the catheter at home. You will stay 1 night in the hospital and you can go home the day after the procedure. You may have pain after the treatment. You will be prescribed painkillers for this. You will return to the hospital 3 days after surgery. During this visit, we will check whether you are able to urinate on your own again. If you urinate well, you can go home without a catheter.

It is important for your recovery that you do not perform heavy physical labor, do not cycle, are not sexually active and do not generate too much abdominal pressure during the first 6 weeks after the operation. There may be erectile dysfunction after treatment. In most cases this is temporary and the erections return after a few weeks to months. Some men experience unwanted urine leakage after the procedure. Usually this is temporary. You may also develop or increase urinary symptoms; you then have to urinate small amounts more often.

Follow-up

You will come for follow-up 3 months after treatment and then after 6 and 12 months, then every six months for up to 3 years and annually for 5 to 10 years. During these visits, it will be checked whether your PSA (prostate specific antigen) does not change and whether the cancer remains away.

Prognosis

The chance that the prostate cancer will return within 10 years is still unknown, because the research is still ongoing. It is probably slightly higher than with surgical prostate removal, or external or internal radiotherapy (scenarios 1 to 4). Should the cancer return, the treatment can be repeated, surgical removal of the prostate, or external or internal radiotherapy can still be performed, with the accompanying risk of side effects as described in scenarios 1 to 4. Also the research into the side effects of this focal treatment is in progress. Therefore, the actual average percentage of side effects may be higher or lower than described below. We can only say for sure in a few years.

The chance that you will die from prostate cancer within 10 years is still unknown, but probably about 1%

Adverse effects

The figures below are for men without the specific complaints before the treatment. In men who already had one or more of the specific complaints before treatment, the complaints persisted or got worse.

- Unwanted urine leakage: about 7% of men experience unwanted urine leakage a year after treatment. 1% use incontinence material.
- Total incontinence: less than 1% of men are totally incontinent one year after treatment.
- Increased urge to urinate: About 7% of men experienced an increased urge to urinate one year after treatment.
- Diarrhea: less than 1% of men suffer from diarrhea one year after treatment.
- Erection problems: about 24% of men have erection problems one year after treatment.

1. Score

On a scale of 0 to 100, what score would you give this scenario?

Dead or worst health imaginable

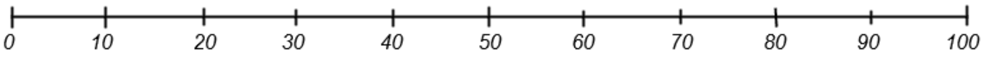

Perfect health

…… points

2. Time

How many years in perfect health, in your opinion, are equivalent to 10 years of life with the above scenario?

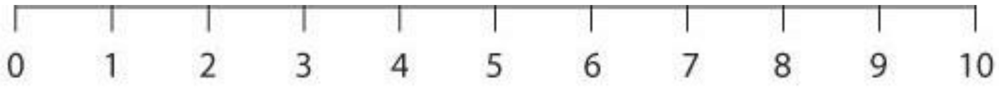

…… years

…… months

Treatment preference

When completing the following question, it is important that you do not look at it from your own situation, but start from the situation described below:

Diagnosis:

You have been told that you have prostate cancer and that you need to undergo treatment. You have not been treated for prostate cancer before. The prostate cancer was discovered after blood tests in which the PSA (prostate specific antigen) was found to be elevated. Subsequently, biopsies (small bites of prostate tissue) were taken from the prostate in which the pathologist found prostate cancer. The tumor is well treatable because tumor tissue is only in the prostate and has not spread, so the chance that you will die from prostate cancer is very small. However, there are various treatment options for which you are eligible, each with its advantages and disadvantages.

Please indicate again which prostate cancer treatment you prefer now after reading the 6 scenarios, assuming you are eligible for all 6 treatments. You can indicate your 1st to 6th choice. You may only use each number once.

…… Scenario 1: Active surveillance of the prostate cancer

…… Scenario 2: Surgical removal of the prostate using the surgical robot (Da Vinci)

…… Scenario 3: External radiotherapy with placing 4 gold markers (1 x 5 millimeters) in the prostate for position determination

…… Scenario 4: MR-Linac guided external radiotherapy with support of MRI scans for position determination during the entire radiotherapy (without placing gold markers in the prostate)

…… Scenario 5: Internal radiotherapy by placing small radioactive seeds in the prostate (LDR "low dose rate" brachytherapy)

…… Scenario 6: Focal therapy in which only the tumor in the prostate is treated via a number of needles and not the entire prostate (Irreversible Electroporation/Nanoknife)
